# Magnitude of benefit of the addition of bevacizumab to first-line chemotherapy for metastatic colorectal cancer: meta-analysis of randomized clinical trials

**DOI:** 10.1186/1756-9966-29-58

**Published:** 2010-05-26

**Authors:** Fotios Loupakis, Emilio Bria, Vanja Vaccaro, Federica Cuppone, Michele Milella, Paolo Carlini, Chiara Cremolini, Lisa Salvatore, Alfredo Falcone, Paola Muti, Isabella Sperduti, Diana Giannarelli, Francesco Cognetti

**Affiliations:** 1 U.O. Oncologia Medica 2 Universitaria, Azienda Ospedaliero-Universitaria Pisana, Pisa, Italy; 2 Department of Medical Oncology, Regina Elena National Cancer Institute, Roma, Italy; 3 Scientiphic Direction, Regina Elena National Cancer Institute, Roma, Italy; 4 Biostatistics, Regina Elena National Cancer Institute, Roma, Italy

## Abstract

**Background:**

Although the addition of bevacizumab to 1^st ^line chemotherapy provides a significant survival benefit for advanced colorectal cancer, the magnitudes of both advantages and toxicities have not been extensively investigated.

**Methods:**

A literature-based meta-analysis was conducted; Hazard Ratios were extracted from randomized trials for primary end-points (Progression Free Survival, PFS, Overall Survival OS). The log of event-based risk ratio were derived for secondary endpoints (objective/partial response rate, ORR/PR; severe hypertension, bleeding and proteinuria). Absolute differences and the number needed to treat/harm (NNT/NNH) were calculated. A meta-regression analysis with clinical predictors and a sensitivity analysis according to the trial phase-design were conducted as well.

**Results:**

Five trials (2,728 pts) were selected. The addition of bevacizumab to 1^st ^line chemotherapy significantly increased both PFS (although with significant heterogeneity) and OS over exclusive chemotherapy by 17.1% and 8.6% (NNT 6 and 12), regardless of the study setting (non significant interaction between phase II and III). The chance to improve PR was significantly increased by 6.5% (NNT 15), with a trend for ORR. The risk of hypertension was significantly increased by 6.2% (NNH 16). According to the meta-regression analysis, female gender and rectal primary site were significant predictors for PFS benefit.

**Conclusions:**

Notwithstanding all the concerns related to costs and the significant HTN risk, the significant outcome improvement provided by bevacizumab in first-line treatment for unselected advanced colorectal cancer patients, should be considered when choosing the appropriate up-front therapy.

## Introduction

The intuition of the relevant role of newly and aberrantly formed blood vessels in driving tumor progression has represented the rational basis to assess the implication of antiangiogenesis as a therapeutic strategy [1].

Preclinical and early clinical successful evidences about the effectiveness of the monoclonal antibody anti-VEGF bevacizumab have been actually confirmed in the large phase III trial AVF2107 [2], whose impressive results have led to the approval of bevacizumab for the treatment of metastatic colorectal cancer (mCRC), in combination with fluoropyrimidine-based chemotherapy.

The introduction of bevacizumab in the daily practice has deeply modified the handling of mCRC patients insomuch as its use has been rapidly and widely adopted as the standard choice for the first-line treatment. The initial design of the pivotal trial planned to randomize patients to receive irinotecan, bolus fluorouracil and leucovorin (IFL) plus placebo, IFL plus bevacizumab, or fluorouracil and leucovorin (5-FU/LV) plus bevacizumab. Final analysis revealed that the addition of bevacizumab to IFL significantly improved OS (primary endpoint, HR: 0.66, p < 0.001), PFS (HR: 0.54, p < 0.001) and RR (44.8% vs 34.8%, p = 0.004).

The planned analysis comparing patients treated with 5-FU/LV plus bevacizumab with those concurrently enrolled in the IFL plus placebo group, revealed no significant differences between arms in terms of OS (HR: 0.82 [0.59-1.15], p = 0.25), PFS (HR: 0.86 [0.60-1.24], p = 0.42) and RR (49% vs 37%, p = 0.66) [3]. The outcome reported in the 5-FU/LV plus bevacizumab arm was consistent with other experiences that explored the use of bevacizumab in combination with 5-FU/LV. In a phase II randomized study, including 104 patients, the combination of bevacizumab with 5-FU/LV resulted in longer time to disease progression (TTP, median TTP: 9.0 months [5.8-10.9] vs 5.2 months [3.5-5.6]) and in better, but not significantly, RR (40% [24-58] vs 17% [7-23]-34) and OS (median OS: 21.5 months [17.3-undetermined] vs 13.8 months [9.1-23]) [4]. Similar results were obtained in another phase II trial, randomizing 209 patients, that were not optimal candidates for irinotecan-containing regimens, to receive 5-FU/LV plus or minus bevacizumab. Patients treated with the antiangiogenic obtained a significantly longer PFS (HR: 0.50 [0.34-0.73], p = 0.0002) and OS, that was the primary endpoint of the study (HR: 0.79 [0.56-1.10], p = 0.160) [5].

Bevacizumab has been also studied in combination with oxaliplatin-based regimens in the NO16966 study, where about 1400 mCRC patients were randomly assigned according to a 2 × 2 design, to receive either FOLFOX or XELOX plus bevacizumab or placebo as first-line treatment [6]. The addition of bevacizumab was associated with significantly longer PFS (HR: 0.83 [0.72-0.95], p = 0.0023), that translated into a trend toward better OS, though not reaching the statistical significance (HR: 0.89 [0.76-1.03], p = 0.077). The magnitude of the effect of bevacizumab seemed less prominent in this experience, when compared with results achieved in the AVF2107 study. The frequent discontinuation of the anti-VEGF together with chemotherapy before disease progression and not for bevacizumab-related toxicity was suggested by authors as a possible explanation for such finding.

On the basis of these results, the choice of bevacizumab in the routine upfront approach to the treatment of mCRC is extremely frequent. In fact, it has been demonstrated relatively safe in association with both irinotecan- [7] and oxaliplatin-containing regimens [8] and its specific toxicity profile appears manageable, by applying appropriate clinical selection criteria [9]. Moreover, differently from the anti-EGFR antibodies, the anti-VEGF may be proposed to all patients, without any molecular restriction.

However, in spite of its wide use, the magnitude of the benefit derived by the addition of bevacizumab to conventional cytotoxics is still controversial. The present literature-based meta-analysis has been conducted with the aim of quantifying the advantage provided in the daily practice by the use of bevacizumab-containing regimens as first-line treatments for mCRC patients.

## Materials and methods

The analysis was conducted following 4 steps: definition of the outcomes (definition of the question the analysis was designed to answer), definition of the trial selection criteria, definition of the search strategy, and a detailed description of the statistical methods used [10,11].

### Outcome definition

The combination of Bevacizumab (BEVA) and chemotherapy was considered as the experimental arm and exclusive chemotherapy as the standard comparator. Analysis was conducted in order to find significant differences in primary and secondary outcomes, according to the reported sequence and definitions in the selected trials. Primary outcomes for the magnitude of the benefit analysis were both Progression Free Survival (PFS, time between randomization and any progression or death for any cause) and Overall Survival (OS, time between randomization and any death). Secondary end-points were: 1) ORR (objective response rate), 2) PR (partial response rate), 3) grade 3-4 hypertension (HTN) rate, 4) grade 3-4 bleeding rate, and 5) grade 3-4 proteinuria rate, if reported in at least 50% of selected trials. The thromboembolic risk was not chosen to be explored because already reported in literature [12]. A sensitivity analysis taking into account the trial design setting (i.e. phase II or phase III) was accomplished.

### Search strategy

Deadline for trial publication and/or presentation was March, 2009. Updates of Randomized Clinical Trials (RCTs) were gathered through Medline (PubMed: http://www.ncbi.nlm.nih.gov/PubMed), ASCO (American Society of Clinical Oncology, http://www.asco.org), ASCO-GI (ASCO Gastrointestinal Symposium), ESMO (European Society for Medical Oncology, http://www.esmo.org), and FECS (Federation of European Cancer Societies, http://www.fecs.be) website searches. Key-words used for searching were: chemotherapy, colorectal cancer, colon, rectal, bevacizumab, targeted, monoclonal antibodies, avastin^®^, review, metanalysis, meta-analysis, pooled analysis, randomized, phase III, phase II, comprehensive review, systematic review. In addition to computer browsing, review and original papers were also scanned in the reference section to look for missing trials. Furthermore, lectures at major meetings (ASCO, ASCO-GI, ESMO, and ECCO) having 'chemotherapy and targeted agents for advanced colorectal cancer' as the topic were checked. No language restrictions were applied.

### Trial identification criteria

All prospective randomized RCTs, either phase II or III, published in peer-reviewed journals or presented at the ASCO, ASCO-GI, ECCO, and ESMO meetings until March 2009, in which previously untreated patients with advanced/metastatic colorectal cancer were prospectively randomized to receive BEVA plus chemotherapy (experimental arm) or chemotherapy alone (control arm), regardless of drug, schedule, dosages, and duration.

### Data extraction

Hazard Ratios (HRs) for primary end-points and the number of events for secondary end-points were extracted; the last trial's available update was considered as the original source. All data were reviewed and separately computed by five investigators (V.V., F.C., D.G., and E.B.).

### Data synthesis

HRs were extracted from each single trial for primary end-points, and the log of relative risk ratio (RR) was estimated for secondary endpoints [13], and 95% Confidence Intervals (CI) were derived [14]. A random-effect model according to the inverse variance and the Mantel-Haenzel method was preferred to the fixed, given the known clinical heterogeneity of trials; a Q-statistic heterogeneity test was used. Absolute benefits for each outcome were calculated (i.e. absolute benefit = exp {HR/RR×log[control survival]} - control survival [15]; modified by Parmar et al [16]). The number of patients needed to treat for one single beneficial patient was determined (NNT: 1/[(Absolute Benefit)/100]) [17]. Results were depicted in all figures as conventional meta-analysis forest plots; a RR < 1.0 indicates fewer events in the experimental arm. In order to find possible correlations between outcome effect and negative prognostic factors (selected among trials' reported factors, i.e. number of patients with: rectal as primary site, female gender and adjuvant treatment), a meta-regression approach was adopted (i.e. regression of the selected predictor on the Log RR of the corresponding outcome). Calculations were accomplished using the SPSS software, version 13.0, and the Comprehensive Meta-Analysis Software, version v. 2.0 (CMA, Biostat, Englewood, NJ, USA).

## Results

### Selected trials

**Figure 1:**
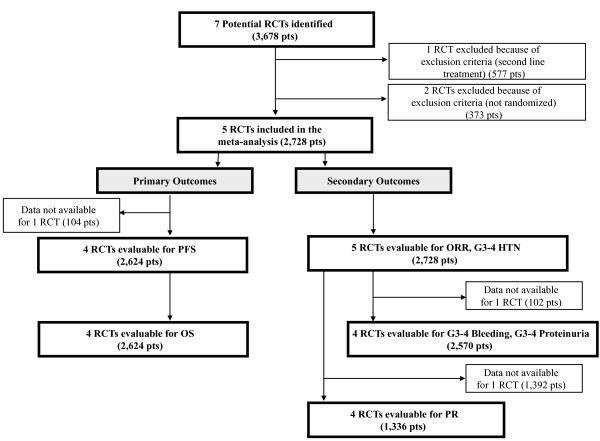
**Outline of the search - Flow diagram**. RCTs: randomized clinical trials; Pts: patients; PFS: progression free survival; OS: overall survival; ORR: overall response rate; PR: partial response rate; HTN: hypertension.

Seven trials (3,678 patients) were identified (Figure 1). One was excluded because of exclusion criteria (i.e. second line treatment) [18], another ruled out owing to not randomized for BEVA assignment [8]. Four RCTs were evaluable for PFS and OS (2,624 patients, data lacking for 104 patients); with regard to secondary outcomes, 5 trials were evaluable for ORR and grade 3-4 HTN analysis (2,728 patients) and 4 trials for grade 3-4 bleeding and proteinuria (2,570 patients). Four trials (1,336 patients) reported data for PR determination, one trial was excluded for lacking data [6]. Trials characteristics are listed in Table 1.

**Table 1:** Trials' characteristics.

Authors	Pts	Phase	Experimental Arm	Primary End-Point	Secondary End-Point	Female (%)	Rectal (%)	Adjuvant (%)
Hurwitz et al[2]	813	III	IFL+Beva	OS	PFS, ORR, DR, QoL	*40%	21%	26%

Hurwitz et al[3]	210	III	FU/LV+Beva	OS	PFS, ORR, DR	39%	29%	-

Saltz et al[6]	1,400	III	FOLFOX/XELOX+Beva	PFS	PFS, OS, RR, DR, TTF	42%	26%	24%

Kabbinavar et al[4]	104	II	FU/LV+Beva	TTP, RR	OS, DR	43%	-	19%

Kabbinavar et al[23]	209	II	FU/LV+Beva	OS	PFS, ORR, DR, QoL	46%	19%	20%

Pts: patients; IFL: irinotecan, 5-fluorouracil, leucovorin; Beva: bevacizumab; OS: overall survival; PFS: progression-free survival; ORR: overall response rate; DR: duration of response; QoL: quality of life; FU: 5-fluorouracil; LV: leucovorin; FOLFOX: 5-fluorouracil, leucovorin, oxaliplatin; XELOX: capecitabine, oxaliplatin; TTF: time to treatment failure; TTP: time to progression.

### Combined Analysis

**Figure 2:**
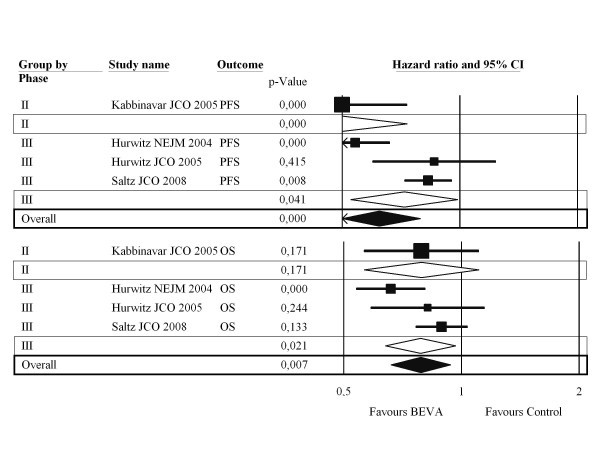
**Combined results according to sensitivity analysis - Primary outcomes**. CI: confidence interval; PFS: progression free survival; OS: overall survival; BEVA: bevacizumab.

Primary Outcomes: the addition of BEVA to chemotherapy significantly increased both PFS (although with significant heterogeneity) and OS over exclusive chemotherapy by 17.1% and 8.6% (Figure 2), respectively, corresponding to 6 and 12 NNT (Table 2). The benefit is obtained regardless of study setting, according to the absence of significant interaction (p = 0.06 and p = 0.93, respectively) between phase II and phase III pooled results.

**Table 2:** Combined efficacy results according to primary and secondary outcomes.

Outcomes	Pts (RCTs)	HR/RR (95% CI)	*p-value*	Het. (*p*)	AD (%)	NNT
PFS	2,624 (4)	0.62 (0.48, 0.69)	*< 0.0001*	*0.001*	17.1	6

OS	2,624 (4)	0.78 (0.66, 0.94)	*0.007*	*0.14*	8.6	12

ORR	2,728 (5)	1.16 (0.97, 1.38)	*0.085*	*0.034*	-	-

PR	1,336 (4)	1.24 (1.06, 1.46)	*0.006*	*0.19*	6.5	15

Pts: patients; RCTs: randomized clinical trials; HR: hazard ratio; RR: relative risk; CI: confidence intervals; Het.: heterogeneity; AD: absolute difference; NNT: number needed to treat; PFS: progression free survival; OS: overall survival; ORR: overall response rate; PR: partial response rate.

### Secondary Outcomes

**Figure 3:**
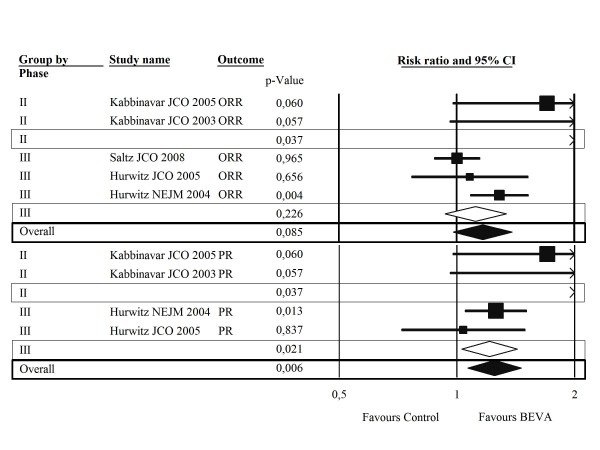
**Combined results according to sensitivity analysis - Secondary outcomes**. CI: confidence interval; ORR: overall response rate; PR: partial response rate; BEVA: bevacizumab.

**Figure 4:**
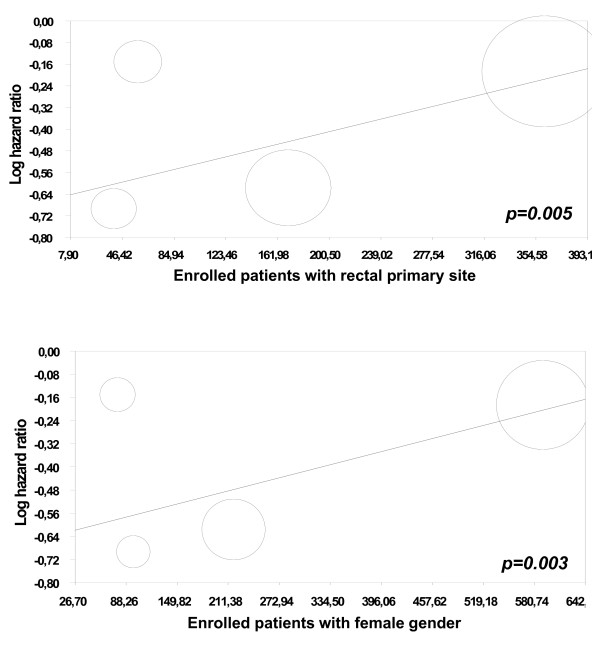
**Significant Predictors for Progression Free Survival (PFS) at the meta-regression analysis**.

the addition of BEVA to chemotherapy significantly increased the chance to achieve PR by 6.5%, which translates into 15 NNT (Table 2); a non-significant heterogenous trend in favour of BEVA is found for ORR rate as well (Figure 3). The risk of hypertension is significantly increased with the addition of BEVA by 6.2%, which corresponds to 16 NNH (Table 3). No significant differences in grade 3-4 bleeding and proteinuria (although a trend against BEVA) were observed by comparing the two arms, without heterogeneity (Table 3). According to the meta-regression analysis, female gender and rectal primary site were significant predictors for PFS benefit (p = 0.003, p = 0.005, Figure 4).

**Table 3:** Combined toxicity (Grade 3-4) results.

Outcomes	Pts (RCTs)	RR (95% CI)	*p-value*	Het. (*p*)	AD (%)	NNH
HTN	2,728 (5)	4.87 (3.12, 7.61)	*< 0.0001*	*0.93*	6.2	16

Bleeding	2,570 (4)	1.72 (0.96, 3.07)	*0.07*	*0.52*	-	-

Proteinuria	2,570 (4)	2.10 (0.64, 6.84)	*0.21*	*0.56*	-	-

Pts: patients; RCTs: randomized clinical trials; RR: relative risk; CI: confidence intervals; Het.: heterogeneity; AD: absolute difference; NNH: number needed to harm; HTN: hypertension.

## Discussion

Combinations of conventional cytotoxics plus BEVA as 1st line treatment for mCRC patients are one of the possible standard options. Given the impressive results of the phase III AVF2107 trial, it seemed almost clear that a biologic agent able to extend median PFS and median OS by more than 4 months, with a 44% reduction of the risk of progression and a 34% reduction of the risk of death (p < 0.001), would have found a wide space in the oncologic practice, considering also its satisfactory toxicity profile. However, such exciting results produced by adding BEVA to the IFL regimen have not been fully confirmed by subsequent trials that tested the addition of the antiangiogenic to other regimens. In particular, the NO16966 study (oxaliplatin based doublets plus or minus BEVA) met its primary endpoint of improving PFS for patients treated with bevacizumab, with a smaller than expected reduction in the risk of progression of 17% (p = 0.0023), but this did not translate in a significant advantage in terms of OS [6]. A plausible explanation for such findings resides in the discontinuation of BEVA - even independently from the occurence of BEVA-related toxicities - before disease progression much more frequently in this study, in comparison to the pivotal trial by Hurwitz et al [6].

Moving from the above reported results it has been hypothesized that the advantage produced by the addition of BEVA in first-line may vary depending on the combination regimen adopted and that it has been more evident with an almost abandoned regimen (IFL). This underlines the importance of meta-analyses trying to estimate the cumulative magnitude of BEVA's effect.

According to the results of the present meta-analysis, the addition of BEVA to first-line chemotherapy regimens (IFL, FOLFOX, XELOX, 5-FU/LV) would provide a significant advantage in terms of both PFS and OS, with an increase of 17,1% and 8,6% respectively, in comparison to exclusive chemotherapy.

On the other hand, BEVA does not seem to allow to achieve an higher rate of response, even if a trend toward significance (p = 0.085) is reported. Such finding is not surprising at all, since it is well known that tumoral shrinkage may represent an inappropriate parameter, in order to appreciate the real benefit provided by antiangiogenic drugs. Such agents are able to exert a clinically meaningful disease control, that translates into a significant improvement of survival, even though not determining an impressive tumor downsizing. This observation acquires a crucial importance in the choice of the best biologic agent (bevacizumab *vs *cetuximab) to be combined with upfront chemotherapy, especially in patients with potentially resectable disease. At the same, it should be considered that anti-EGFRs are not active in *KRAS *mutant patients while the only data from a phase III random study looking at response rate with BEVA in a subgroup of *KRAS *wild-type patients demonstrated a response rate of 60% for IFL plus the anti-VEGF [19]. This does not differ too much from the 59.3% obtained in the CRYSTAL trial adding cetuximab to FOLFIRI for *KRAS *wild-type patients [20]. Only head-to-head ongoing phase III random trials will address this question.

As it regards the toxicity profile, it is confirmed the relatively safe use of BEVA, as already suggested by BEAT [9] and BRiTE registers [21], that included about 4000 patients, treated with the anti-VEGF in the clinical practice. In the present metanalysis the addition of BEVA significantly increased the risk of hypertension by 6.2%, while no significant differences in grade 3-4 bleeding and proteinuria were observed.

According to the our meta-regression analysis, female gender and rectal primary site were significant predictors for PFS benefit: we do not have any biological or clinical explanation for such unexpected finding. Future studies should be conducted for confirming these results and therefore to drive reliable hypothesis.

According to our results, the addition of BEVA to first-line chemotherapy seems to improve treatment's efficacy in an overall population, selected on the basis of the inclusion criteria of gathered trials, that tended to exclude patients prone to experience BEVA-related toxicities because of their cardiovascular comorbidities or bleeding diatheses. Despite that, from a clinical perspective, the identification of molecular predictors of benefit from the antiangiogenic drug could be extremely useful to refine patients' selection and to improve the cost-effectiveness ratio [22]. In fact, on the one hand, this step forward could allow to avoid the harmful cost of unnecessary and potentially life-threatening toxicities to patients with poor chances to achieve benefit from the anti-VEGF antibody. On the other hand, the magnitude of the advantage provided by the addition of BEVA to chemotherapy would be certainly more extensive in a better selected population [22].

The above reported observations acquire an even more crucial importance, considering the current possibility to administer both the anti-VEGF bevacizumab and the anti-EGFR cetuximab - for which only patients with *KRAS *wild-type disease are candidate - in the first-line approach to mCRC, but not at the same time. The detrimental effect of the double inhibition binds the oncologist to face an unavoidable point of decision for the handling of *KRAS *wild type patients and only the availability of new markers of benefit may help to define the best strategy for each patient.

## Competing interests

The authors declare that they have no competing interests.

## Authors' contributions

FL, EB, VV and FC participated in the conception and the design of the analysis; EB, FC, IS and DG performed the statistical analysis. FL, EB, VV, CC, and LS revised the whole writing process. MM, PC, AF, PM, DG and FC participated in its design and coordination. All authors read and approved the final manuscript
